# Supplementary material for: Moderate Constraint Facilitates Association and Force-Dependent Dissociation of HA-CD44 Complex
Source: Int J Mol Sci. 2023 Jan 23;24(3):2243. doi: 10.3390/ijms24032243 (PMC9917194; doi:10.3390/ijms24032243)
Supplement: Supplementary file 1 [file ijms-24-02243-s001.zip › Supplementary Materials.pdf]

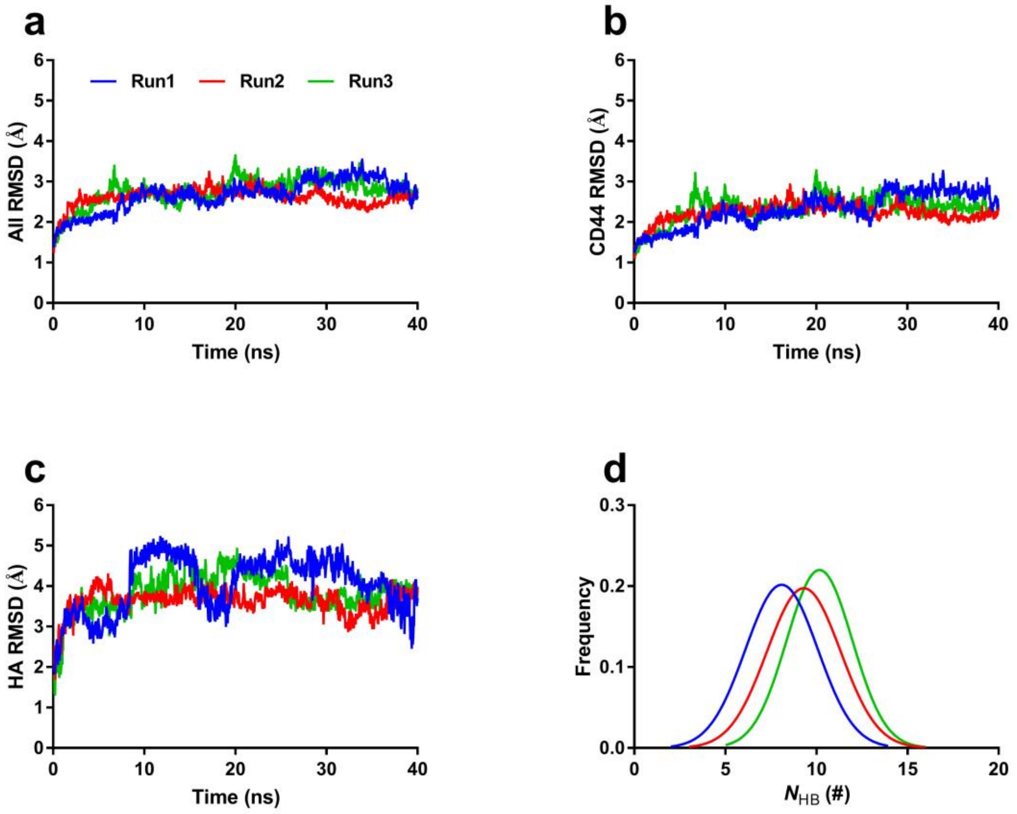

Supporting Information Figure S1. The structure and stability of the complex of CD44 with HA during 40 ns equilibrium. The time course of RMSD of (**a**) the complex of the CD44 with HA, (**b**) CD44 alone and (**c**) HA alone. Each RMSD data comes from three independent runs. (**d**) The distribution of H-bond (N_{HB}) during 40 ns equilibrium. The data for NHB frequency sampled from simulation time of 40 ns were fitted to the Gaussian distributions.

Table S1. The interaction probabilities of significant H-bonds on the binding site of CD44/HA during the three equilibriums.

Numbers	CD44 residue	HA residue	Interaction probabilities P(%)			Mean P(%)
			Run1	Run2	Run3	
1	ARG155	1181	23.27	—	16.78	13.35
2	TYR34	1181	—	21.65	—	7.22
3	ARG155	1180	88.45	92.35	79.20	86.67
4	GLU79	1180	—	34.77	10.15	14.97
5	ARG82	1179	—	—	40.90	13.63
6	ARG82	1178	40.60	59.60	56.90	52.37
7	CYS81	1178	31.20	91.10	90.10	70.80
8	ARG45	1178	52.99	69.14	79.06	67.06
9	ILE100	1177	48.70	68.30	65.20	60.73
10	TYR46	1177	59.70	76.60	69.80	68.70
11	ARG45	1177	—	16.10	21.40	12.50
12	ARG45	1176	—	62.80	69.23	44.01
13	TYR119	1175	40.78	42.60	71.93	51.77
14	ASN115	1175	76.19	57.81	79.88	71.29
15	THR113	1175	53.47	—	—	17.82

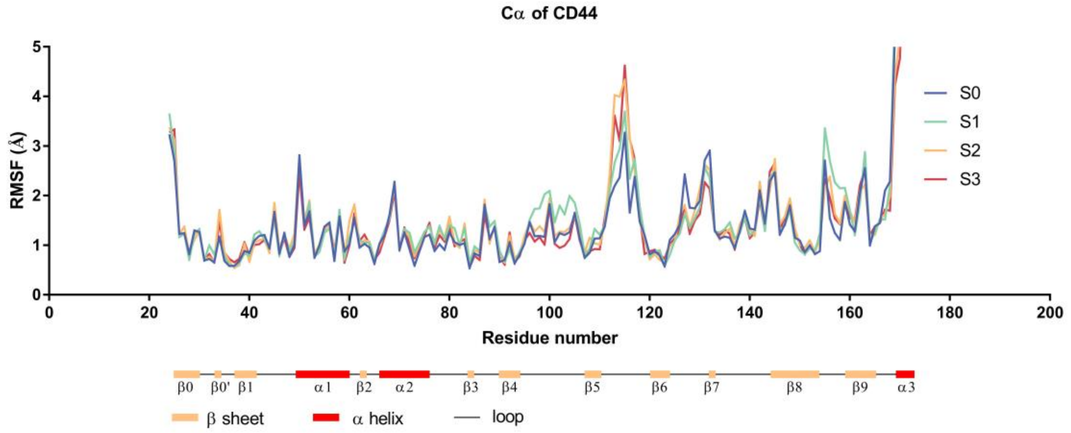

Supporting Information Figure S2. The Cα-RMSF of residues of bound CD44 on different constraints in system S0, S1, S2 and S3. Each date was the mean from three independent run over 100 ns for each given HA constraint manner.

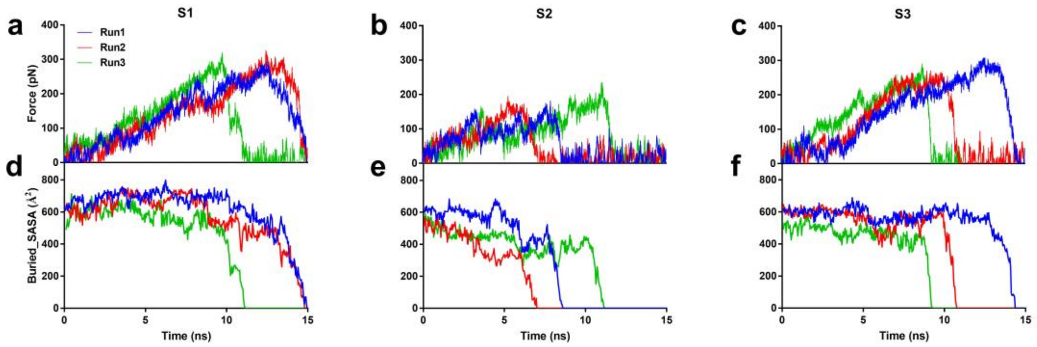

Figure S3. (**a-c**) The time course of loading force and (**d-f**) Buried-SASA on the complex for three force-ramp SMD simulation under different HA constraints.

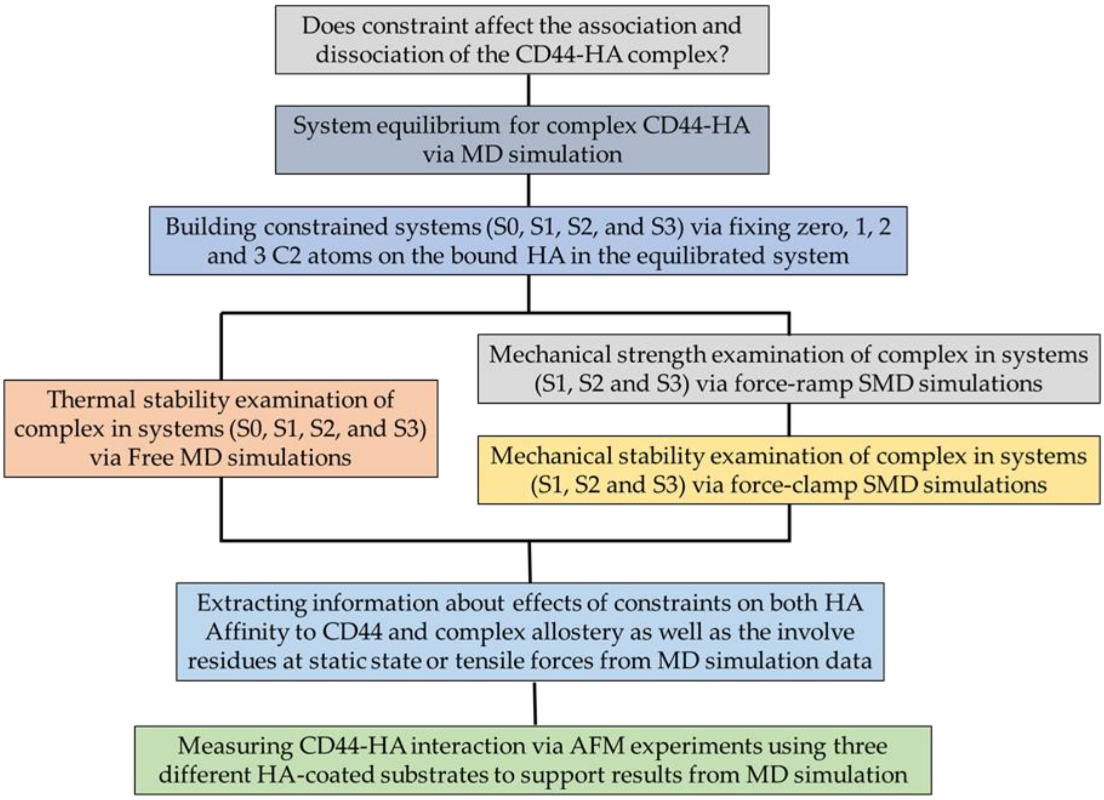

Figure S4. The flow diagram for study mechanical constraints on CD44-HA interaction via MD simulation and AFM experiments.

Supporting Information Video S1. Video of the dissociation process of system S1 under force-ramp mode.

Supporting Information Video S2. Video of the dissociation process of system S2 under force-ramp mode.

Supporting Information Video S3. Video of the dissociation process of system S3 under force-ramp mode.
